# Electro-casting for Superior Gas Separation Membrane Performance and Manufacturing

**DOI:** 10.1021/acsami.3c14742

**Published:** 2023-11-22

**Authors:** Sharifah H. Alkandari, Bernardo Castro-Dominguez

**Affiliations:** † Department of Chemical Engineering, University of Bath, Bath BA2 7AY, United Kingdom; ‡ Centre for Digital Manufacturing and Design (dMaDe), University of Bath, Bath BA2 7AY, United Kingdom

**Keywords:** gas separation membranes, electric field crystallization, carbon capture, ionic liquids, composite membrane, electro-casting

## Abstract

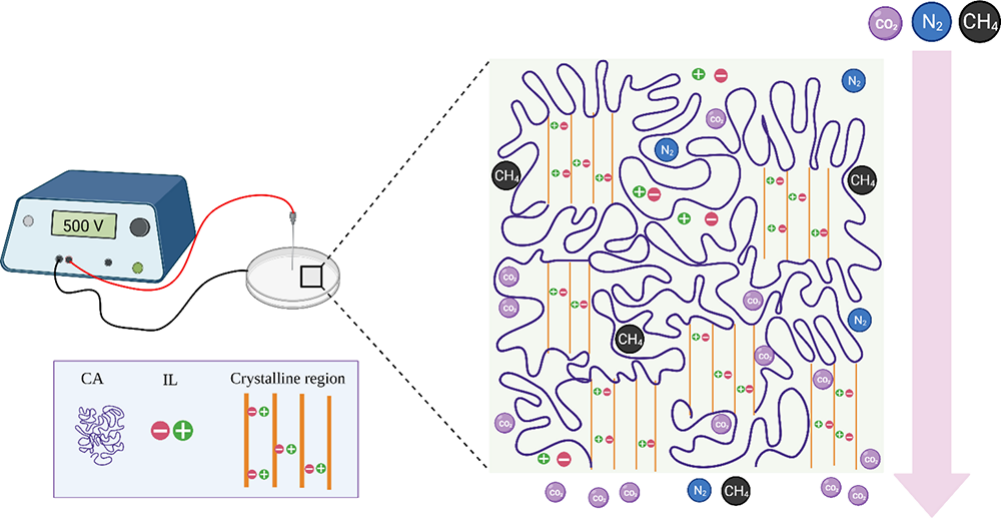

Gas separation polymer membranes play a pivotal role in various industrial processes including carbon capture and hydrogen production. However, the inherent trade-off between permeability and selectivity coupled with challenges in membrane manufacturing has hindered their widespread industrial deployment. To address the permselectivity challenges, researchers have explored increasingly complex polymers, composite systems, and other materials. In this study, we introduce a novel membrane manufacturing technique called “electro-casting” that not only enables efficient membrane fabrication but also enhances the trade-off of traditional polymer-based membranes. We fabricated cellulose acetate (CA) membranes embedded with 1-ethyl-3-methyl imidazolium via electro-casting and performed a comparative analysis of structural, morphological, and gas transport characteristics against membranes made via conventional casting techniques. We discovered that electro-casted membranes exhibited a unique crystalline structure, surface topology that induced a remarkable 200% improvement in CO_2_/N_2_ selectivity and a 110% increase in CO_2_/CH_4_ selectivity. The electric field generated during the manufacturing process played a crucial role in altering the supramolecular structure of the polymer, thereby increasing the separation properties of the membranes as well as their thermal and mechanical features. Electro-casting induced a polymer crystallization effect that disrupted the permeability-selectivity trade-off observed in conventional membranes, while producing highly stable membranes. Moreover, the simplicity of this manufacturing method and its significant impact on membrane properties have the potential to accelerate the deployment of gas separation membranes, facilitating the transition toward a NetZero chemical industry.

## 1 Introduction

Polymer membrane technologies used for gas separation processes–such as carbon capture, hydrogen purification, and oxygen enrichment–are desirable due to their low maintenance requirements, high energy efficiency, sustainable traits, and small equipment size.^1^ Although gas separation membranes are regarded a key technology to achieve NetZero and to mitigate global warming, there are many challenges that remain to be addressed such as plasticization, poor thermal stability, and the trade-off between selectivity and permeability. Indeed, optimizing the performance of gas separation membranes requires a comprehensive understanding and control of the various factors that influence their transport properties, including the molecular structure, morphology, and crystalline characteristics of the polymer materials employed. On a parallel track, polymer crystallization, as a fundamental process in polymer science, has attracted substantial attention. The crystalline structure formed during polymer solidification significantly impacts the mechanical, thermal, and transport properties of the resulting materials. Researchers have explored numerous strategies to control and manipulate polymer crystallization, aiming to enhance the performance and functionality of polymeric systems.

Despite their independent trajectories, the fields of gas separation membranes and polymer crystallization exhibit intriguing overlaps and complementarity. Recent studies have started to unravel the intricate relationship between the crystallization behavior of polymers and the transport properties of gas separation membranes. Understanding and controlling this synergistic interplay can offer profound insights for tailoring the performance of gas separation membranes by harnessing the unique characteristics of polymer crystallization. For instance, Kloos et al. aligned polymerizable liquid crystals (LCs) to demonstrate the effect of supramolecular orientation on gas separation performance.^2^ They found that polymer alignment increased the ideal selectivity of He/N_2_ by 36-fold and 21-fold for CO_2_/N_2_. The team proposed that molecular order reduced the free volume elements in the membrane, which hindered the diffusion of gases with larger kinetic diameters. Beyond enhancing the selectivity of membranes, higher crystallinity in polymers reduces chain mobility and restrains the CO_2_-induced swelling effect of chains, effectively mitigating the pressure plasticization.^3^

Polymer scientists have used various techniques to control the crystallization of polymers, such as (i) molecular design, which includes selecting polymers and copolymers prone to order; (ii) polymer additives, which includes the use of nucleating agents and chain modifiers; (iii) thermal processing, which includes cooling rate control and annealing; and (iv) processing techniques, which includes mechanical stretching and solvent-induced crystallization. However, they are not transferable for membrane manufacturing or change the intrinsic chemistry of the polymer, thus affecting their gas separation properties.

This work shows, for the first time, that electro-casting, a process that uses electric field forces to orientate materials and generate crystalline structures, can be exploited to induce supramolecular control during membrane manufacturing.^4−6^ Therefore, to assess the reach of this technique, cellulose acetate (CA) was chosen for the proof-of-concept as it is one of the most common commercial membrane materials. Moreover, to ensure responses of the CA polymeric chains under electric fields and therefore control the crystallinity of the polymer; ionic liquids (ILs) are used as the solvent media. Budkov et al. mathematically demonstrated that a polymer chain immersed in dielectric solvents shows conformational responses when subject to an external electric field.^7^ Beyond these important traits, ILs were chosen as they are environmentally friendly, room temperature liquid salts with negligible vapor pressure, high recyclability, nonflammable nature, and excellent chemical and thermal stability.^8^ ILs in combination with polymers have shown enhanced CO_2_ separation performances due to active diffusivity. For instance, Klepic et al. blended membranes using poly(vinyl alcohol) (PVA) and 1-ethyl-3-methylimidazolium dicyanamide [EMIM][DCA] for CO_2_/H_2_ gas separation. The study found that increasing the amount of IL in the blend membrane resulted in increases in gas permeability and H_2_/CO_2_ selectivity. The team reported that with more than 20 wt % of IL loading, the membrane was more permeable to CO_2_ and at an IL concentration of 53 wt %, they were able to overcome the CO_2_/H_2_ Robeson (2008) upper bound.^9^

Although there is evidence across the literature that IL incorporation in polymer membranes improves the separation performance, the impact of applying electric fields and tuning the crystal structure of the membranes remains to be explored. Consequently, this study aims at exploring the effect of electro-casting on CA doped with 1-ethyl-3-methylimidazolium dicyanamide [EMIM][DCA] and the corresponding effect on CO_2_/CH_4_ and CO_2_/N_2_ separation performance.

## 2 Methods and Materials

### 2.1 Materials and Membrane Preparation

#### Selection of Materials

**Figure 1:**
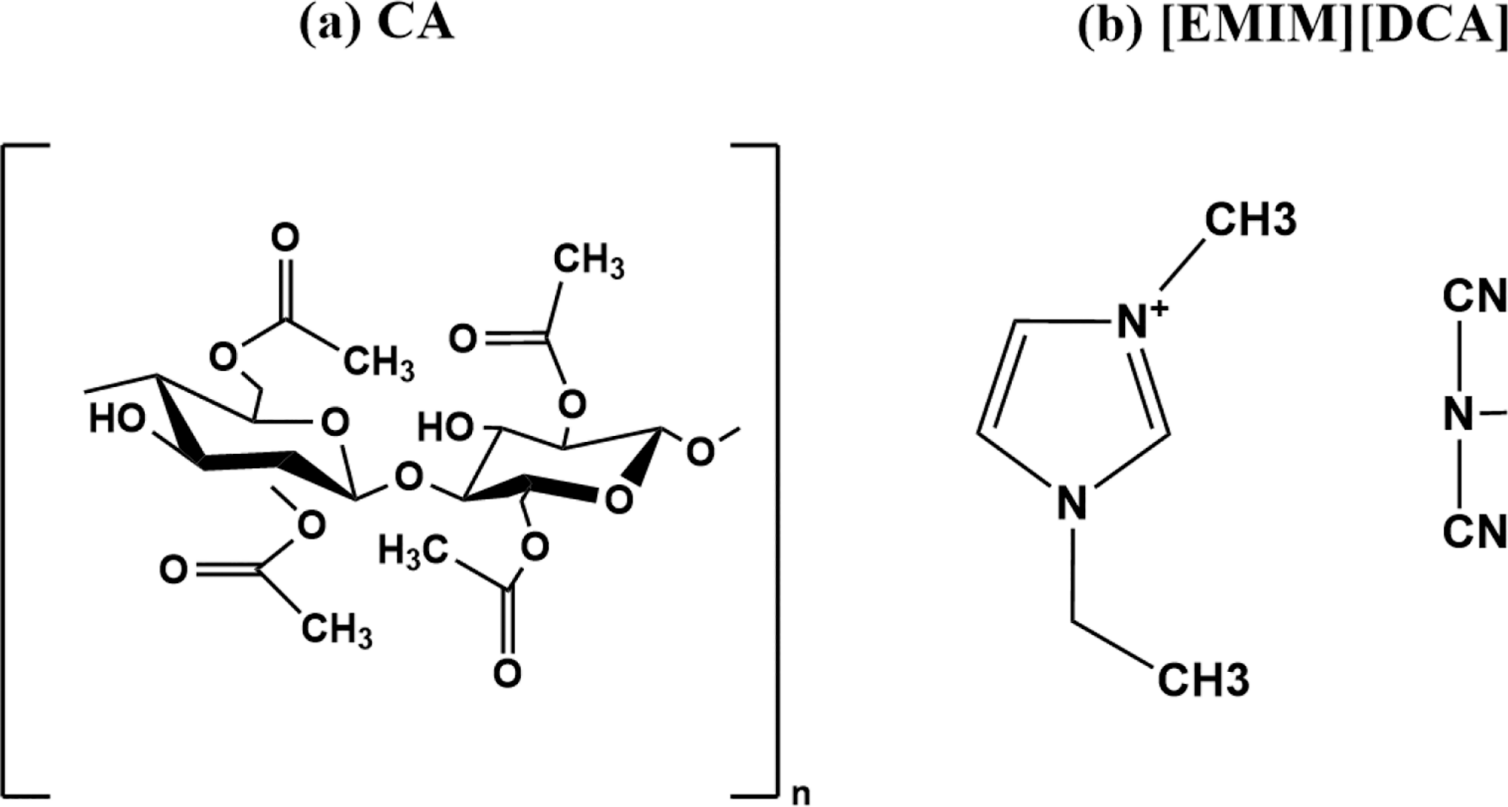
Chemical structures of (a) cellulose acetate (CA) and (b) [EMIM][DCA].

Room-temperature ionic liquid (RTIL), 1-ethyl-3-methylimidazolium dicyanamide [EMIM][DCA] with 98% purity, was purchased from Thermo Fisher Scientific, UK. [EMIM][DCA] was chosen as the ideal RTIL for its high conductivity (2.0 × 10^–3^ S/m)^10^ and its ability to maintain consistent CO_2_ permeance and gas selectivity across a wide range of CO_2_ and CH_4_ partial pressures, ranging from 0 to 207 kPa and 0 to 300 kPa, respectively.^11^ Acetone, *N*,*N*-dimethylacetamide (DMAc), and cellulose acetate (CA) with a number-average molecular weight of 3.0 × 10^4^ Da were supplied by Aldrich Chemical Co. Inc. in a fine, dry, and free-flowing powder form. All the materials and solvents used in this study was used as received. Figure 1 shows the chemical structures of CA and [EMIM][DCA], while Table 1 outlines some of their physicochemical properties.

**Table 1:** Physical Properties of Materials

materials	melting point (°C)	viscosity (×10^–3^ cP)	density (g/cm^3^)	thermal decomposition (°C)
CA	220–290		1.3	300–400
[EMIM][DCA]	–21	21	1.1	275

#### Membrane Casting

The CA-[EMIM][DCA] composite membrane was fabricated by a solution casting and electro-casting process as follows. The casting solutions were prepared by combining a specified amount of polymer (15% w/w of solution), solvent (acetone and DMAc in a 2:1 ratio), and IL ([EMIM][DCA]). The IL concentration was varied from 0% to 40% (w/w of polymer). A 20 g solution was prepared for synthesizing the membrane and stirred overnight. Prior to casting, the solution was sonicated for 30 min and degassed for 4 h. Subsequently, the solution was poured into a leveled Petri dish, left overnight to air-dry in controlled conditions, and then placed in a vacuum oven at 60 °C overnight. Please note that the literature has shown loads as high as 81 wt %,^12^ thus these membranes have a conservative amount of IL. Moreover, it was found that for our membranes, once the IL reached a load of 40 wt.%, the membranes were unstable and hence displayed structural flaws and a tendency for the IL to leak. Consequently, the maximum load of IL in these membranes was of 40 wt.%

#### Membrane Electro-casting

**Figure 2:**
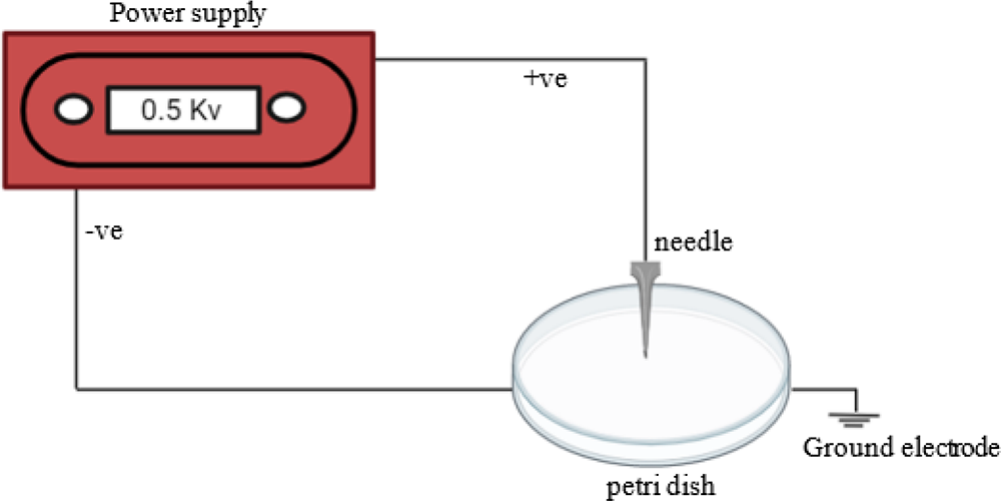
Illustration of CA-[EMIM][DCA] composite membrane electro-casting procedure.

The casting solution was poured into a stainless-steel Petri dish placed between two perpendicular electrodes (one vertically from the needle and the other horizontally attached to the Petri dish) and allowed to dry at room temperature overnight. While drying, direct electric potential of 500 V was supplied as depicted in Figure 2. Note that this voltage is lower than that used in conventional electrospinning systems, which can react ∼20 kV. The membranes were heated overnight in a vacuum oven at 60 °C. Note that this investigation was performed at a fixed voltage and varied [EMIM][DCA] loading to examine the effect of the IL quantity on the composite CA-[EMIM][DCA] membrane. Table 2 shows all membranes synthesized in this study and their nomenclature.

**Table 2:** Membranes Synthesized in This Study and Nomenclature

membrane nomenclature	IL load (wt %)	synthesis method
pure CA	0	casting
CA-IL10	10	casting
CA-IL20	20	casting
CA-IL30	30	casting
CA-IL40	40	casting
CA-IL10-e	10	electro-casting
CA-IL20-e	20	electro-casting
CA-IL30-e	30	electro-casting
CA-IL40-e	40	electro-casting

### 2.2 Membrane Characterization

#### Structural Characterization

The supramolecular structure of the membranes was assessed using Powder X-ray diffraction (PXRD) and wide-angle X-ray diffraction (WAXD). The membranes were scanned on a STOE STADI P double setup operated by using pure Cu Kα1 radiation (wavelength of 1.54060 Å). The quantitative phase analysis and crystal structure analysis were performed using WinXPOW software. The WAXD instrument utilized is SAXS point 2.0 by Anton-Paar equipped with copper source (1.542 and 0.7107 Å, 50 W) and a 2D EIGER R series Hybrid Photon Counting (HPC) detector. Moreover, the surface and cross-sectional morphology of the membranes were analyzed using a variable pressure scanning electron microscopy (SEM, SU3900, Hitachi, Japan), incorporated with energy-dispersive X-ray spectroscopy (EDX).

#### Chemical and Thermal Characterization

The chemical structure analysis was carried out to determine the functional group details of the membranes using Fourier transform infrared spectroscopy (FTIR, Spectrum 100TM PerkinElmer USA), packaged with total reflectance cell ranging from 4000 to 650 cm^–1^. Prior to the actual sample analysis, a baseline scan was run in transmission mode, at a spectra resolution of 4 cm^–1^, and the spectra were recorded for the total reflectance cell range. Moreover, the thermal properties of the membranes were analyzed using a Differential Scanning Calorimeter (DSC, TA Instruments Q2000) that was equipped with an intercooler refrigeration system. The sample was then subjected to a cycle of heating–cooling–heating within a temperature range of −50 °C to +200 °C under a nitrogen flow rate of 20 mL/min at a heating rate of 10 °C per minute. To minimize the baseline curvature, an empty holder of aluminum was used as a reference in the alternative sample holder of the DSC. Prior to the analysis, the composite membrane samples were vacuum-dried overnight at 40 °C to remove any water that may have been adsorbed due to the IL’s hygroscopic nature. Simultaneously, thermogravimetric analysis (TGA) was conducted using a (TGA, TA Instruments Q-500), wherein the sample (approximately 10 mg) was heated from a temperature of 20 to 800 °C at the rate of 10 °C min^–1^, under an argon flow rate of 60 mL/min. The degradation temperature, expressed in terms of weight loss as a function of temperature, was measured at the first derivative peak of the TGA curve, using the Setsoft 2000 thermal analysis software.

#### Mechanical Properties

The mechanical properties of the membranes were analyzed using a tensile testing INSTRON (3369, England) instrument at room temperature. The membranes were pulled or stretched at a fixed speed of 10 mm/min, a head load of 5 kN, and a grip length of 5 cm. Each test was repeated at least three times. This method was used to determine the tensile stress, tensile strain at break, and tensile modulus.

#### Gas Separation Measurements

Single-gas (CO_2_, CH_4_, and N_2_) permeation measurement was conducted using a constant volume/variable pressure time-lag apparatus at a feed pressure of 3 bar and 25 °C as well as in variable pressure values. The gas permeability was calculated from the steady-state rate of pressure increase at a fixed downstream volume. All details are described in the Supporting Information (S1). It is important to acknowledge that our evaluation of the membranes was limited to room temperature conditions. This deliberate choice was made because room temperature has been found to facilitate improved gas separation across a wider range and facilitate the effortless integration of these membranes into different industrial contexts, thereby obviating the necessity for complex temperature regulation protocols. However, further investigation is necessary to assess the impact of temperature variations on the efficacy of gas separation in membranes produced by using this novel approach.

#### Finite Element Methods for Electric Field Modeling

The electro-casting process was simulated in a 2D finite element method (FEM) model using COMSOL Multiphysics to assess the electric field strength across the membrane. The physics used in this model were “electrostatics”, which is used to describe the electric field caused by nonmoving charges. It assumes that the membrane is a dielectric, where charges can be displaced by an external electric field, resulting in induced electric dipoles. Please refer to the Supporting Information for more details (S5).

## 3 Results

**Figure 3:**
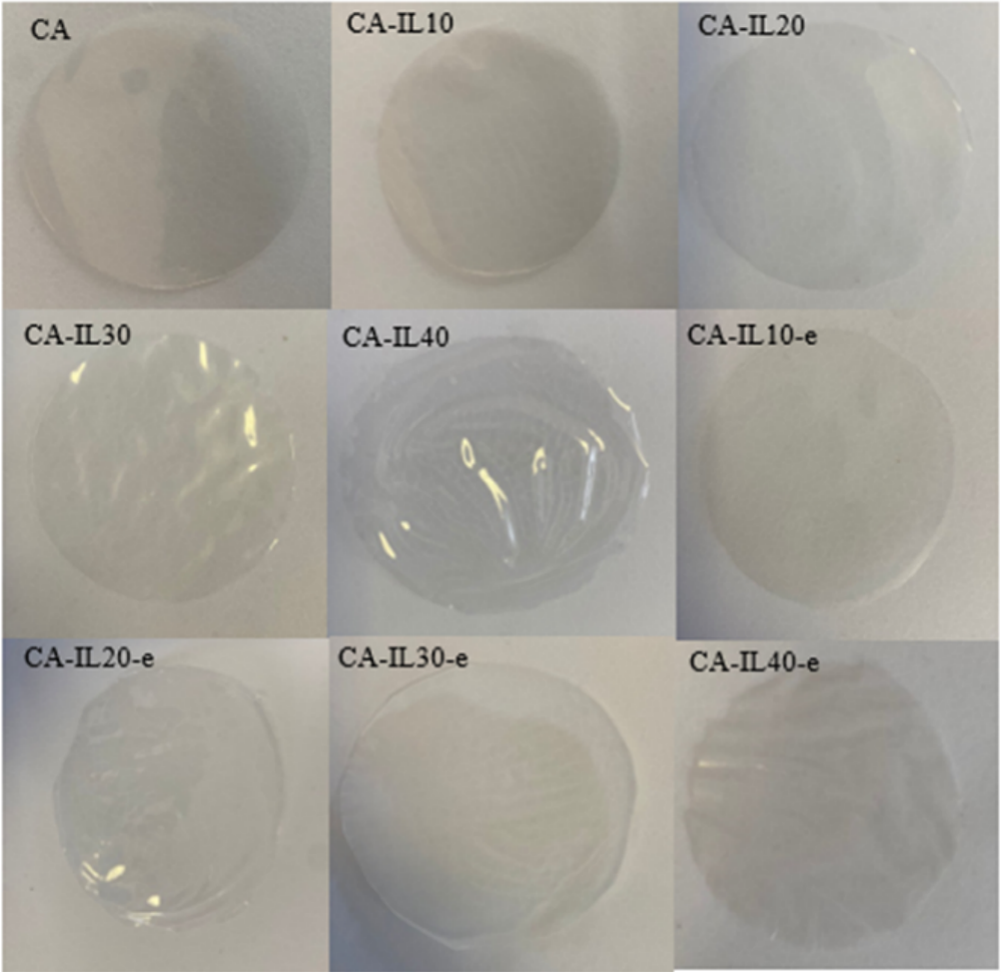
Samples of membranes fabricated with various IL loads and techniques.

The membranes were fabricated via casting and electro-casting techniques to comparatively assess the effect of the electric field. All membranes generated in this work were mechanically robust, self-supported membranes regardless of the ratio of the CA polymer and [EMIM][DCA]. All the membranes prepared in this manner were either transparent or slightly hazy, similar to the pristine polymer as shown in Figure 3. Exudation of ILs on the surface of the membranes was not present in any of the membranes developed in this work, indicating that they were stable. These are all further supported by the FTIR results which showed the presence of both the IL and the polymer (see Figure S1). As a result, all membranes were subjected to characterization.

### 3.1 Thermal and Chemical Analysis of the Membranes

In general, all composite CA-IL membranes exhibited a chemical composition that combines the features of the pristine CA and pure [EMIM][DCA], as evidenced by their absorption bands on the FTIR analysis, which is presented in the Supporting Information (Figure S1). It was found that with increasing ILs weight percentage, the appearance of the IL becomes significant on both sides of the composite membranes (Figure S1a). These findings suggest that the IL is uniformly dispersed within the membranes as there is no discernible difference between the FTIR spectra of the top and bottom sides. The FTIR spectra of the composite membranes manufactured via electro-casting displayed no new functional groups and was identical as those manufactured via casting (Figure S1b). This implies that the high voltage applied during casting did not induce any reactivity of the precursor materials.

**Figure 4:**
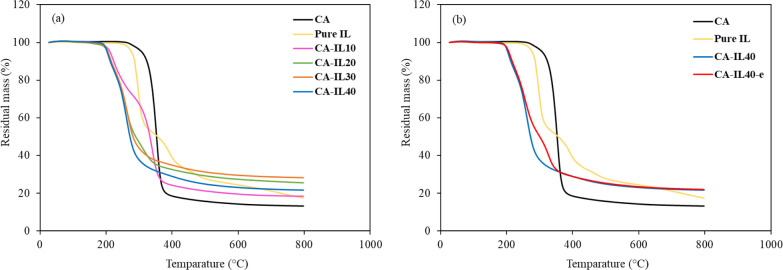
TGA analysis for: (a) pure CA, pure IL, and CA-IL loading of 10 to 40 wt %; (b) pure CA, pure IL, and CA-IL40-e.

Thermal analysis via TGA was conducted to assess the degradation and stability of the membranes. Figure 4a shows the effect of incorporation of the IL into the CA matrix. In general, the thermal features of the composite membranes proportionally combine the features of the CA and [EMIM][DCA] as evidenced by the residual weight loss of the membranes. The gradual increase of residual weight lost at higher IL loads provides further evidence of the presence of the IL in the polymer matrix. These findings align with previous research reported elsewhere.^13^ Moreover, it appears that the presence of the IL promoted weight loss at reduced temperatures, suggesting that the IL acts as polymer plasticizer. Figure 4b shows the effect of electro-casting on a composite membrane with 40 wt % IL. The electro-casted membrane shows enhancement in thermal stability compared to the conventional casted membrane. Finally, DSC results, shown in Figure S5, revealed that electro-casted membranes showed a slight increase in the glass transition temperature from 36 to 42 °C. Please refer to the Supporting Information for more details (S2).

### 3.2 Membrane Crystallinity

**Figure 5:**
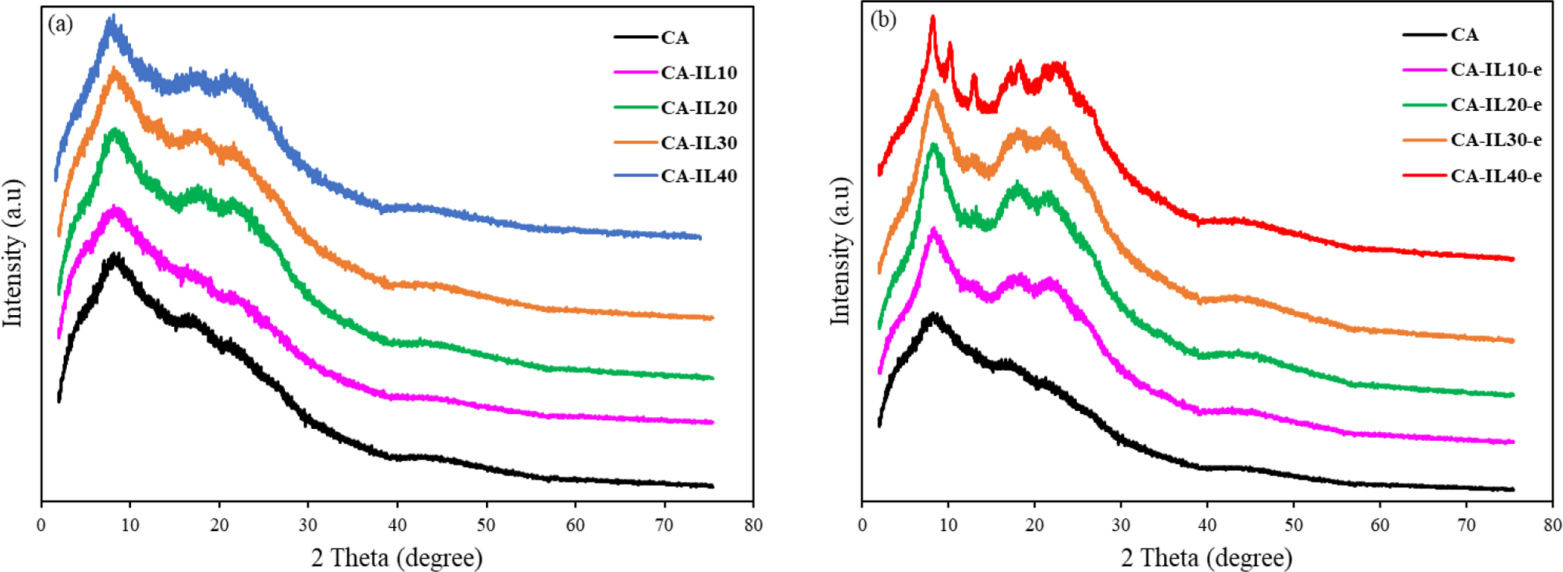
XRD spectra of (a) pure CA and CA-ILs without an electric field; (b) pure CA and CA-ILs with an electric field.

Figure 5a,b shows the XRD patterns of all membranes synthesized via casting and electro-casting, respectively. The XRD of the pure CA membranes shows a broad curve spanning from 5 to 75° along with three main peaks at 8, 17, and 21°. This broad curve is typical of noncrystalline natural polymers and confirms its amorphous nature.^14−16^ Incorporating IL in the range of 10–40 wt % did not exhibit significant effects on the crystalline structure of the membranes. However, higher IL concentrations led to more pronounced peaks at 17° and 21°, corresponding to the (101) and (020) planes, as depicted in Figure 5a. This behavior aligns with findings in the literature regarding poly(vinyl alcohol)-ionic liquid membranes (PVA-IL).^9^ Notably, the *d* spacing of CA-IL membranes closely resembled that of pure CA membranes. Nevertheless, the presence of the IL noticeably impacted the structural integrity of the CA chains, resulting in broader halos.

To assess the membranes’ structural response to electric field strengths, various tests were conducted. It was observed that electric field strengths below 300 V/mm had no discernible effect on the architecture of the CA-IL membranes. However, when subjected to 500 V/mm or higher, the membranes exhibited chain alignment along the direction of the applied field (refer to Supporting Information Figure S4). Furthermore, membranes subjected to a 500 V/mm electric field displayed more prominent peaks at 17° and 21°, along with the emergence of new peaks at 10° and 12°. These results suggest that higher IL concentrations, combined with the influence of an electric field, contribute to inducing supramolecular order within the CA chains. It is noteworthy that no chemical reactions occurred between the CA and the IL, as evidenced by the FTIR spectra provided in the Supporting Information.

The concentration of the IL has a notable impact on the electrical and transport properties of an IL-containing solution, including its responsiveness to an applied electric field. Elevated IL concentrations typically result in heightened ionic conductivity and increased ion mobility, which can lead to more pronounced phenomena when subjected to an electric field.^17^ This augmented ion conductivity and ion mobility can induce discernible effects, such as a greater molecular alignment of the polymer.^18^ As depicted in Figure 5b, all membranes exhibited increased crystallinity; however, in the case of CA-IL40-e, a substantial enhancement is observed compared with membranes with lower IL concentrations. This remark suggests that a concentration of 40 wt % IL is necessary to enable ion migration to the extent where it can exert a notable influence on CA.

**Figure 6:**
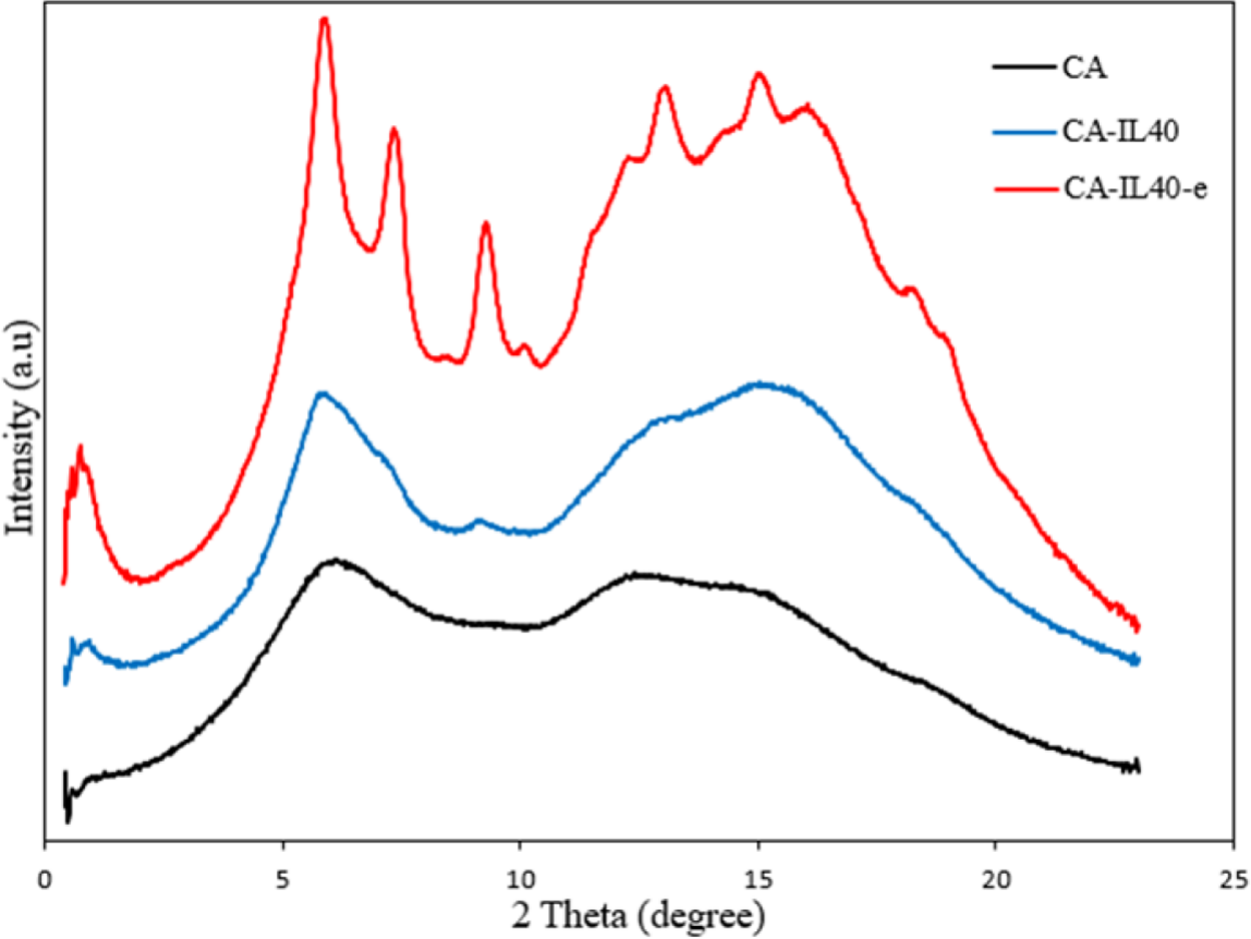
WAXD spectra of pure CA, CA-IL40, and CA-IL40-e.

The degree of crystallinity was further explored via WAXD for pure CA, CA-IL40, and CA-IL40-e membranes, as shown in Figure 6. By comparatively assessing these membranes, compared to pure CA, CA-IL-40 displayed emerging peaks at 7.0 and 14.0 nm^–1^. However, when the membrane was electrocasted (red), the WAXD pattern displayed prominent peaks at 8.5 and 10.5 nm^–1^, which are not noticed in both the pure CA and CA-IL40 membranes. In addition, in order to assess the uniformity of crystallinity across the membrane, samples were collected at various points on the membrane. The results, depicted in Figure.S3b, revealed a consistent characteristic peak at the respective intervals of the membrane, providing further confirmation of the uniformity achieved through the electro-casting process.

### 3.3 Membrane Morphology

**Figure 7:**
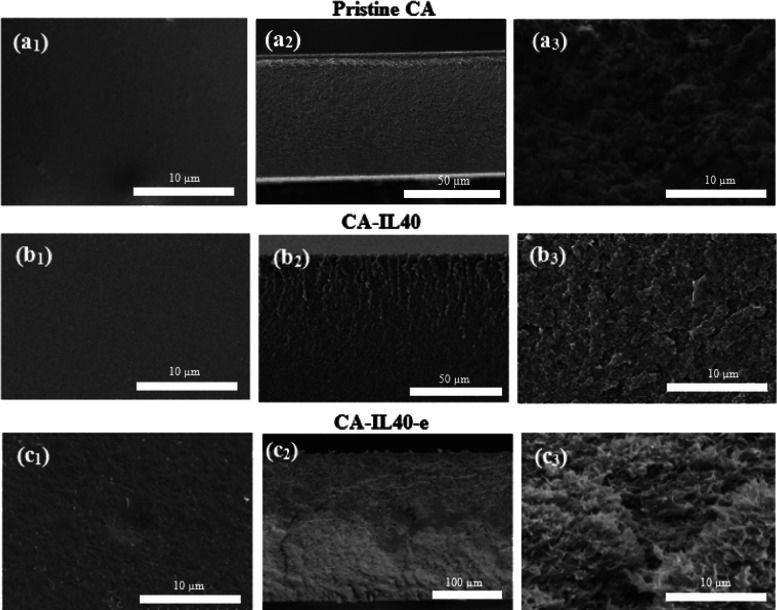
SEM images for: (a_1_) pristine CA surface, (a_2_) pristine CA cross- section, (a_3_) pristine CA zoomed cross-section; (b_1_) CA-IL40 surface, (b_2_) CA-IL40 cross-sectional low magnification, (b_3_) CA-IL40 cross-sectional high magnification; (c_1_) CA-IL40-e surface, (c_2_) CA-IL40-e cross-sectional low magnification, (c_3_) CA-IL40-e cross-sectional high magnification.

SEM images of all membranes were obtained and used to assess the surface morphology (Figure 7). Figure 7_1_a, Figure 7_2_a, and Figure 7_3_a depict the surface, cross-section, and zoomed cross-sectional SEM images, respectively, of the pure CA membrane. The surface morphology of the pure CA membrane was observed to be defect-free, smooth, and flat, which is a commonly reported characteristic feature in the literature.^15,19,20^ The morphology of the composite membranes at various IL loadings without the application of an electric field, shown in Figure 7_1_b, Figure 7_2_b, and Figure 7_3_b, revealed a smooth and uniform surface. Notably, there were no discernible differences in surface images between the pristine CA and CA-ILs membranes at various loads. The SEM images of CA-IL40 displaying a smooth surface suggest that indeed, CA and [EMIM][DCA] exhibited compatibility within the examined range of ILs fabricated.

Figure 7c_1_, 7c_2_, and 7c_3_ shows that when an electric field was applied during membrane casting, there was a significant impact on the morphologies of CA-IL40-e membranes, displaying a surface that appeared rough with a hairy-like topology. Even so, the membranes retained their nonporous, dense structure. The thickness of the membranes ranged from 150 to 200 μm, and no indication of pore formation or surface defects was observed on the cross-section of the membranes.

### 3.4 Membrane Mechanical Properties

The results for tensile stress, tensile strain at break, and tensile modulus of the pristine CA, CA-40IL, and CA-40IL-e membranes are presented in Table 3. Significant differences were seen in the tensile modulus, which is a measure of a material’s rigidity and ability to resist deformation when subjected to tension. The CA membrane in its original state had a significantly higher tensile modulus of 917 MPa, especially when compared to that of the membranes that contained IL. The presence of the IL led to a significant decrease in the tensile modulus, principally ascribed to the attenuation of polymer–polymer interactions. In contrast, the CA-40IL-e membranes had a comparatively elevated tensile modulus, increasing from 119 to 167 MPa, as compared to the CA-40IL membranes. The observed rise in magnitude can be ascribed to the augmentation of polymer–polymer interactions and therefore an increase in crystallinity, as depicted in Figure 5.

**Table 3:** Mechanical Properties of the Membranes

sample	tensile modulus (MPa)	tensile stress at break (MPa)	elongation at break (%)
CA	917.64	16.87	3.80
CA-IL40	119.11	3.00	13.10
CA-IL40-e	167.69	4.15	10.40

A comparable pattern was noted in relation to the tensile strength at break, a crucial characteristic that signifies a membrane’s ability to endure maximal tensile loads prior to failure. Pristine CA demonstrated the greatest tensile strength at the point of fracture, with CA-40IL-e membranes ranking second. Once again, the CA-40IL material exhibited the lowest tensile strength at the point of fracture. Finally, the membranes containing IL exhibited enhanced elongation at the point of fracture, indicating superior stretchability and deformation properties in comparison to the original CA membrane. This effect has been thoroughly shown across the literature^18^

The IL acted as a plasticizer, decreasing the molecular interaction between chains and therefore reducing the mechanical features of CA. Notably, the imposition of an external electric field during membrane synthesis increased the crystallinity of CA and therefore enhanced the mechanical properties lost due to the presence of the IL.

### 3.5 Gas Permeation Analysis

**Figure 8:**
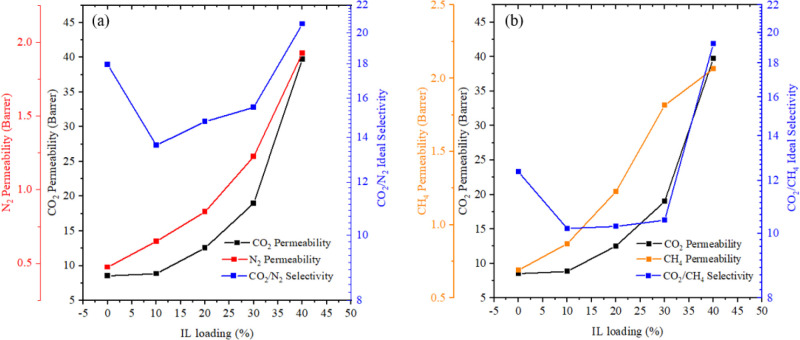
Permeability and ideal gas selectivity of conventional casted CA membranes with various IL loadings for pure gases: (a) CO_2_, N_2_, and CO_2_/N_2_ selectivity; (b) CO_2_, CH_4_, and CO_2_/CH_4_ selectivity.

Figure 8 displays the gas permeation characteristics of both pristine CA and composite CA-IL membranes with varying IL loadings. The investigation assesses the influence of the IL content on the gas permeation of pure gases, namely, CO_2_, CH_4_, and N_2_. This analysis was conducted at room temperature (25 °C) under fixed and variable feed pressure of 3 bar. For a more detailed exploration of the impact of varying feed pressure on membrane performance, refer to the Supporting Information (S3).

The results presented in these figures reveal that gas permeability increases proportionally with an increase in the IL loading. For instance, in Figure 8a, the pristine CA membrane exhibited the lowest permeability for both CO_2_ and N_2_ gases (8.5 and 0.5 Barrer, respectively), whereas the maximum permeability values for both gases (39.7 and 1.9 Barrer, respectively) were observed at the highest IL loading of 40 wt.%. Likewise, Figure 8b demonstrates that in the absence of IL, the gas permeability values for CO_2_ and CH_4_ were the lowest at 8.5 and 0.7 Barrer, respectively. However, at a 40 wt % IL loading, these permeability values increased to 39.7 and 2.1 Barrer, respectively.

These figures also indicate that the ideal selectivity of gases (CO_2_/N_2_ and CO_2_/CH_4_) increased as the IL loading increased. Notably, the composite CA-IL membrane exhibited the maximum selectivity at a 40 wt % IL loading, suggesting that the IL preferentially enhances the permeability for CO_2_. The overall trend in the selectivity plot suggests a direct correlation between IL loading and ideal selectivity, consistent with previous research findings.^13,21^

**Figure 9:**
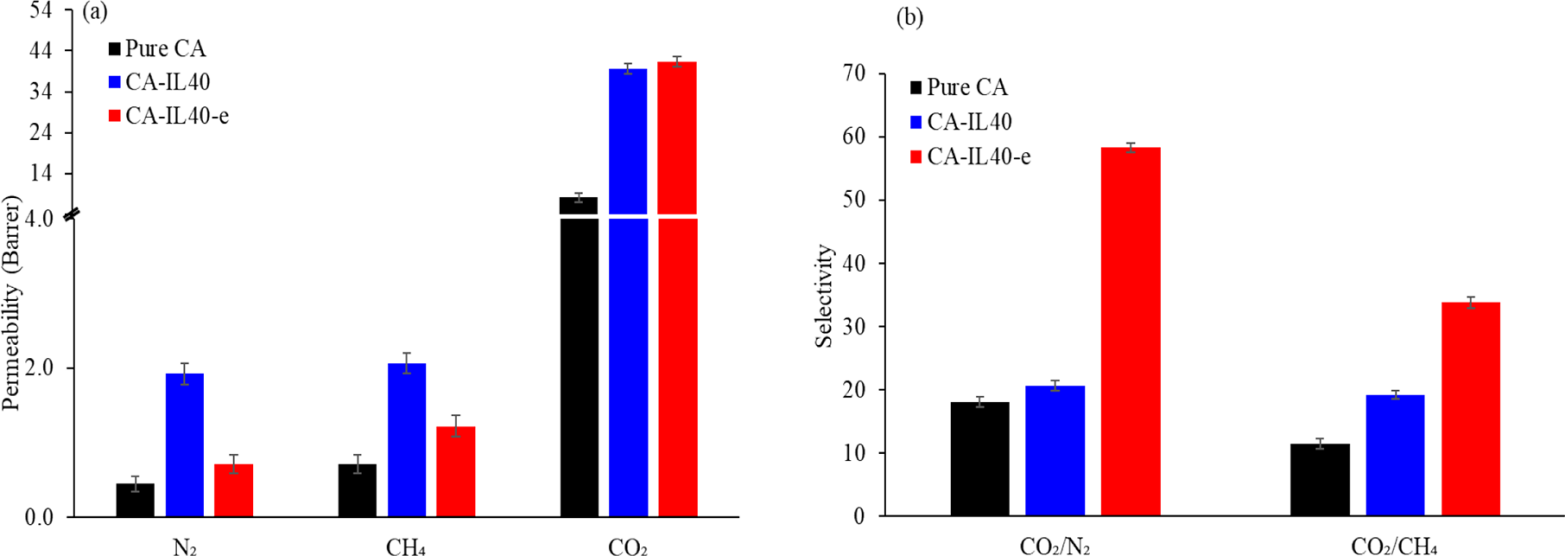
Comparison of (a) the gas permeability of pure CA and CA-IL membranes fabricated with and without an electric field and (b) the ideal gas selectivity of pure CA and CA-IL membranes fabricated with and without an electric field.

The effect of the electric field on the gas permeability and ideal selectivity of composite CA-ILs membranes (CA-IL40-e) was investigated and compared against those fabricated without (CA-IL40). We explored the effect of the electric field on various membranes fabricated at different IL loads (10 to 30 wt %) and the results are presented in Table S1. These studies revealed that the permeability and selectivity of these membranes did not show any changes in their overall performance. However, once the IL content reacted 40 wt %, the membranes boosted their performance. Figure 9 shows that the application of an electric field in the fabrication of the composite CA-ILs membrane at high IL loads resulted in a significant change in the membrane separation performance. For N_2_ and CH_4_, the gas permeability of CA-IL40-e is lower than CA-IL40. However, the CO_2_ permeability remained constant. By maintaining a constant CO_2_ permeability and reducing the N_2_ and CH_4_ permeability, the CA-IL40-e membrane displayed a CO_2_/N_2_ and CO_2_/CH_4_ ideal selectivity significantly higher than those of membranes fabricated without an electric field.

**Figure 10:**
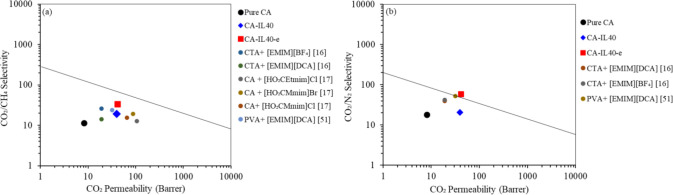
Comparison of the separation performance of pure CA, CA-IL40 and CA-IL40-e membranes field on Robeson Upper bound for (a) CO_2_/N_2_ and (b) for CO_2_/CH_4_.

The separation performances of the membranes developed in this work were comparatively assessed against those from other research studies and presented in the Robeson upper bond shown in Figure 10. In Figure 10a, the CO_2_/N_2_ selectivity versus CO_2_ permeability is shown in the Robeson Upper bound plot. The upper bound line on a Robeson plot provides a general estimate of the maximum selectivity achievable for a particular set of pure-gas permeability values in polymer-based materials. This serves as a useful guideline for the development of new membrane materials.^22,23^ The results indicate that the membrane fabricated with an electric field application exhibited better separation performance than those fabricated without an electric field despite having the same IL loading. Additionally, the permselectivity of CA-IL40-e was slightly above the 1991 Robeson upper bound line, indicating that the membrane fabrication techniques employed in this study could be a potential high-performing approach for manufacturing membranes intended for CO_2_/N_2_ separation. Furthermore, the composite CA-IL membrane showed superior performance compared with the pristine CA membrane. Similarly, Figure 10b depicts the Robeson upper bound plot for CO_2_/CH_4_ for the various membranes fabricated in this study. The results follow the same trend as discussed previously, with the membrane fabricated with a higher IL loading and an electric field application exhibiting higher permselectivity than those fabricated without an electric field at the same IL loading. The pristine CA membrane showed the lowest permselectivity, as shown in Figure 10a. These observations are consistent with those reported by other authors who investigated the performance of ionic liquids doped polymeric membranes.^13,16^ Overall, for both the CO_2_/N_2_ and the CO_2_/CH_4_ separation, the doping of CA with ILs resulted in an improvement of the separation properties, bringing them closer to the Robeson upper bound line. This reveals the effectiveness of this in enhancing CA-based membrane materials for gas separation using membranes.

### 3.6 Stability Test of the Membranes

Being CA a glassy polymer, it has the tendency for physical aging, which can diminish the gas permeability over time.^24,25^ This aging was confirmed by long-term permeation tests on the CA-IL40-e membrane after 480 and 5040 h. Figure.S8 shows a reduction in the relative permeability of all gases, which was calculated as the ratio of a measured value to the initial permeability at the start of the experiment. Most notably, there was an 8% reduction in CO_2_ permeability within the initial 480 h, and only a 4% decline was preserved between 480 and 5040 h. On the other hand, N_2_ and CH_4_ exhibited a more significant decline in permeability than CO_2_ permeability, increasing the selectivity of the membrane to 36, and 61 for CO_2_/CH_4_ and CO_2_/N_2_, respectively. The reduction in permeance is caused by a higher polymer crystallinity or densification of the membrane, an effect which has been well-documented in the literature^26^ and which has been confirmed by the XRD patters of the aged membranes, shown in Figure S9 in the Supporting Information.

Glassy polymers maintain a nonequilibrium shape when their temperature is below the glass transition temperature (*T*_g_). Hence, the polymer chains exhibit the ability to gradually reach a condition of thermodynamic equilibrium, where the polymer chains attempt to move to a lower energy state, causing a reduction in free volume (See Figure.S10). This relaxation process leads to the densification or crystallization of CA, resulting in reduced permeability. Nevertheless, the CA-IL40-e membrane remains in equilibrium even when the temperature is below the *T*_g_ because of the presence of the IL, which adds to the electrostatic interaction between the positively and negatively charged ionic components within the polymer structure. The robust electrostatic contacts have the capacity to effectively immobilize the polymer chains, impeding their natural thermodynamic rearrangement. Despite being below its *T*_g_ and theoretically in a nonequilibrium state, the polymer can exhibit equilibrium-like behavior due to the presence of these interactions. In other words, the possibility of chains undergoing rearrangement diminishes over time, potentially reducing or eliminating the consequences associated with physical aging.^27^ This assessment suggests that the CA-IL40-e membrane is stable and exhibits superior resilience to physical aging compared to pristine glassy polymers.^24^

## 4 Discussion

The polymer chains found in conventional cast CA membranes are tightly packed and intertwined, resulting in an amorphous structure, as indicated by the XRD pattern shown in Figure 5a. Various factors have the potential to influence the density of membrane packing and its overall structure. These factors include processing condition,^28^ polymer molecular weight^29^ and post treatment methods.^30^ This supramolecular structure plays a crucial role in determining the separation properties of the membranes, such as gas permeability and selectivity.^31^

In conventional polymer membranes, the transport mechanism of gases is solution-diffusion. This means that gas molecules are initially adsorbed onto the membrane’s surface and then dissolved into the polymer matrix. Subsequently, the gas molecules diffuse from areas of high concentration to areas of low concentration until they reach the other side of the membrane. The diffusion of gas molecules relies on the presence of the available free volume within the membrane, which is established by the spacing between the polymer chains. The ability of gas molecules to move freely is influenced by factors such as their molecular size, shape, and the mobility of the polymer chains.^32,33^

On the other hand, the capability of gases to dissolve or be absorbed by the polymer is determined by factors like the interactions of the gas with the polymer chains and the condensability of the gases.^34^ The diffusivity of CO_2_ in the CA membrane is higher than N_2_ and CH_4_. This enhanced diffusivity is due to the smaller molecular size of CO_2_, which enables them to move easily through the free volume in the polymer matrix.^35,36^

**Figure 11:**
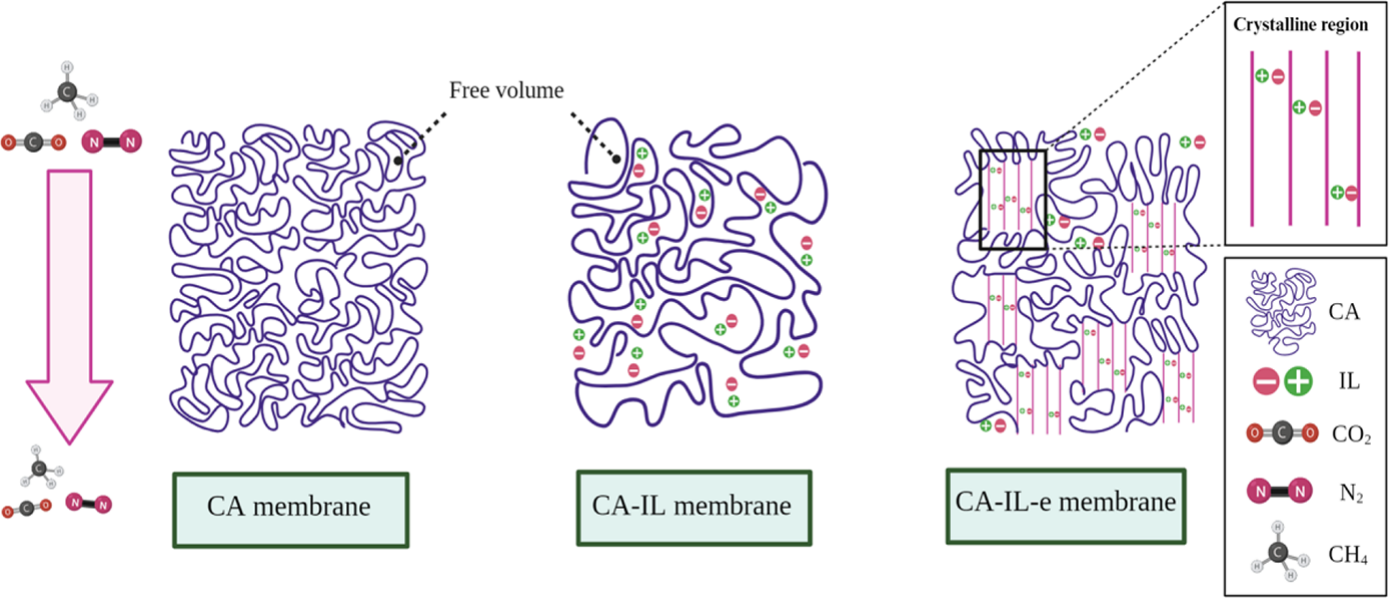
Schematic of gas permeation through the membranes.

The addition of ILs to CA membranes induces a gentle polymer plasticization process by diluting the polymer matrix, consequently enhancing the mobility of the polymer chains.^37^ This promotes easier chain rupture and weakens the intermolecular forces within the polymer matrix, rendering the membrane more vulnerable to thermal degradation, as depicted in Figure 4a by the TGA. Even so, this plasticization provides benefits to gas permeation. Figure 11 shows a schematic that illustrates the gas transport of the CA-IL membranes. While the transport mechanism remains based on solution-diffusion, the enhanced flexibility of the polymer chains facilitates the passage of gases through the membrane. Given that the ILs exhibit a more fractional free volume compared to the pristine CA membrane, gas molecules are able to diffuse, as illustrated by previous authors.^38−40^ Moreover, the incorporation of the IL facilitated the absorption of CO_2_ in the polymer, leading to an increase in both the CO_2_ permeability and gas selectivity. The augmentation in permeability is comparatively more noteworthy for CO_2_ due to its superior diffusivity and solubility.^39,41,42^ This can be observed by an enhanced CO_2_ selectivity, which can be attributed to the highly delocalized nature of its anions^43^ and minimal bonding with N_2_ and CH_4_.^16,44^

By incorporation of an electric field into the fabrication process of CA-IL membranes, notable structural changes were observed, as illustrated in Figure 7c3. The XRD patterns revealed an enhanced level of crystallinity within the membrane (Figures 5b and 6.) The electric field was applied by means of a centrally positioned needle, which facilitated the creation of a uniform crystalline membrane. Interestingly, the influence of this needle, acting as a single high-intensity electric field point, extended throughout the polymer. As shown in Figure S11 in the Supporting Information, which presents a CFD model, the electric field exhibited high intensity in the vicinity of the needle and gradually diminished radially. Despite this, an investigation into the crystalline homogeneity of the composite membrane was conducted by sampling at various intervals (1, 10, 25, and 40 mm) on the surface, as shown in (Figure S3a). The XRD analysis (Figure S3b) confirmed the spatial uniformity of crystallinity within the membrane. The authors propose that the single electric field point is sufficient to induce crystal nucleation, which subsequently propagates across the membrane surface.

Based on the FTIR results shown in Figure S1b, the absence of any electrochemical reactions during the membrane fabrication process indicates that the observed enhancement in selectivity of pair gases (CO_2_/N_2_ and CO_2_/CH_4_) is not attributed to any reaction, but rather a change in the physical state of the membrane. However, the application of an electric field can influence the interactions between the ions and cations by altering the charge distribution and inducing ion movement within the liquid.^45,46^ Positively charged cations are attracted to the negatively charged cathode, while negatively charged anions are attracted to the positively charged anode. This attraction between the charges of cations and anions in the ILs, caused by the electric field, pulls them toward opposite sides, resulting in a more ordered arrangement of ILs within the polymer matrix.^47,48^ This effect could lead to a better alignment of polymer chains and a reduction in tortuosity, facilitating the more efficient diffusion of CO_2_. Even so, it is well-known that the presence of polymer crystallinity decreases membrane permeability, as gas transport primarily occurs in the amorphous regions.^16,49,50^

As the membrane undergoes increased crystallization, the molecular chains align more closely, resulting in a reduction in available free volume and a decrease in the interchain spacing. This tighter arrangement of CA chains typically restricts the movement of gases with larger kinetic diameters, such as N_2_ and CH_4_. However, our findings reveal an intriguing exception, with CO_2_. Surprisingly, the permeability of CO_2_ remains consistent in the CA-IL40-e and CA-IL40 membranes. The observed atypical occurrence can be ascribed to the CO_2_ absorption by the IL cation. According to our hypothesis, upon the solubilization of CO_2_, a CO_2_–cation complex is created, which has the potential to interfere with the ionic interactions responsible for the crystal structure of the CA-IL40-e membrane. This CO_2_–IL complex creates a temporal plasticization effect that enables the diffusion of CO_2_ through the crystalline membrane in a similar fashion to those of the amorphous CA-IL40 membrane. It is noteworthy to mention that upon desorption of the CO_2_ molecule into the permeate, the membrane undergoes a restoration of its original supramolecular structure.^16^

The results indicate a simple and effective technique for manufacturing membranes through the utilization of the electric field during the casting process. This approach successfully shifts the separation properties closer to the Robeson upper bound line, as demonstrated in Figure 10, resulting in a substantial enhancement for CO_2_ selectivity, with an increase on CO_2_/N_2_ selectivity of 200%, while the CO_2_/CH_4_ selectivity experiences a remarkable enhancement of 110%. The electro-casted membranes exhibited enhanced thermal stability and improved some of the mechanical properties, including higher tensile modulus and tensile stress at break. These improvements can be attributed to the increased molecular chain order in these membranes. It is worth noting that IL-polymer membranes shown in this work are stable and, in fact, displayed a higher ideal selectivity after 5040 h.

## Conclusions

In this study, cellulose acetate (CA)-based composite membranes, incorporating 1-ethyl-3-methyl imidazolium [EMIM][DCA] ionic liquid, were successfully fabricated using the electro-casting technique. The application of a high electric field during membrane solution casting led to the rearrangement of cations and anions within the ionic liquid, resulting in better alignment of the polymer chains and increased membrane crystallinity. This structural modification caused by the electric field had a significant impact on the thermal and mechanical properties and membrane’s separation performance. While the enhancement in membrane crystallinity led to a decrease in N_2_ and CH_4_ gas permeability, the permeability of CO_2_ remained constant. This effect induced a substantially higher selectivity of CO_2_ over N_2_ and CH_4_ compared with membranes cast without the electric field. The inclusion of 40 wt % ionic liquid in the electro-casted membranes resulted in a remarkable 200% increase in CO_2_/N_2_ selectivity and a notable 110% boost in CO_2_/CH_4_ selectivity. Moreover, electro-casted membranes showed good stability after 5040 h, displaying a mild permeance reduction, but an increase in their ideal selectivity. This work demonstrates the potential of the electro-casting technique as a novel manufacturing approach for developing enhanced composite membranes for gas separation. The ability to control membrane structure and properties through the application of an electric field opens new possibilities for tailoring membrane performance in various separation applications.

## Supporting Information Available

Characterization of the CA matrix and IL; XRD patterns, FT-IR spectra, SEM of CA-IL membranes, detailed section on membrane separation performance, membrane performance with different feed pressure, membrane stability figures, membrane thermal analysis and electric field modeling. (PDF)
