# Supplementary material for: Electro-casting for Superior Gas Separation Membrane Performance and Manufacturing
Source: ACS Appl Mater Interfaces. 2023 Nov 22;15(48):56600–11. doi: 10.1021/acsami.3c14742 (PMC10711710; doi:10.1021/acsami.3c14742)
Supplement: Supplementary file 1 — am3c14742_si_001.pdf [file am3c14742_si_001.pdf]

Supporting Information

Electro-casting for Superior Gas Separation Membrane Performance and Manufacturing

Sharifah H. Alkandari[a], and Bernardo Castro-Dominguez[a,b,*]

[a]*Department of Chemical Engineering, University of Bath, Bath BA2 7AY, United Kingdom.*
[b]*Centre for Digital Manufacturing and Design (dMaDe), University of Bath, Bath BA2 7AY, United Kingdom.*

* **Corresponding author:** Email: bcd28@bath.ac.uk; Office Number: +441225384946

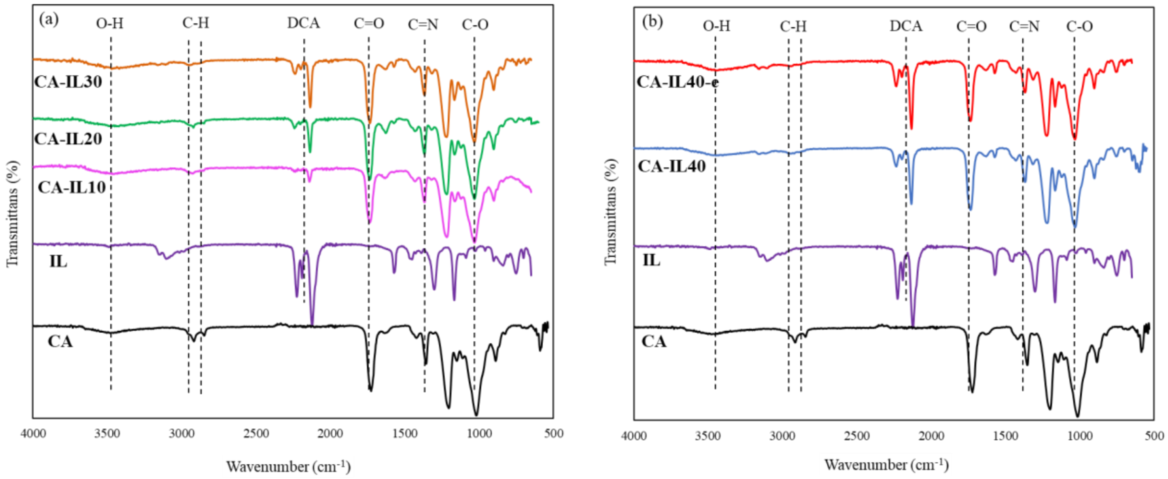

Fig. S1: FTIR Spectrum of (a) pristine CA and CA-IL for different IL loading, and (b) CA+ 40% IL with and without an electric field.

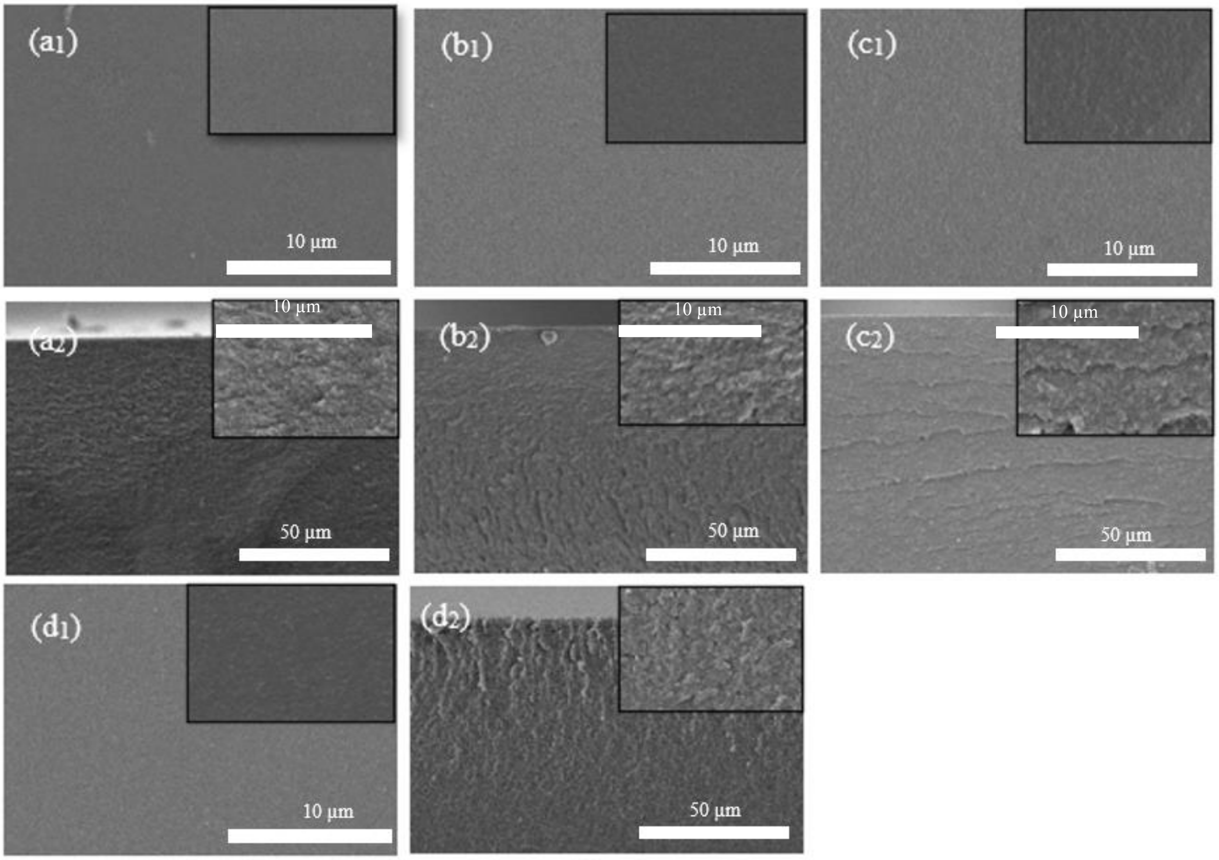

Fig. S2: SEM images for: (a_1) CA-IL-10 surface (a_2) CA-IL-10 cross section, (b_1) CA-IL-20 surface, (b_2) CA-IL-20 cross section, (c_1) CA-IL-30 surface, (c_2) CA-IL-30 cross section, (d_1) CA-IL-40 surface, (d_2) CA-IL-40 cross section. The small square box represents the high magnification.

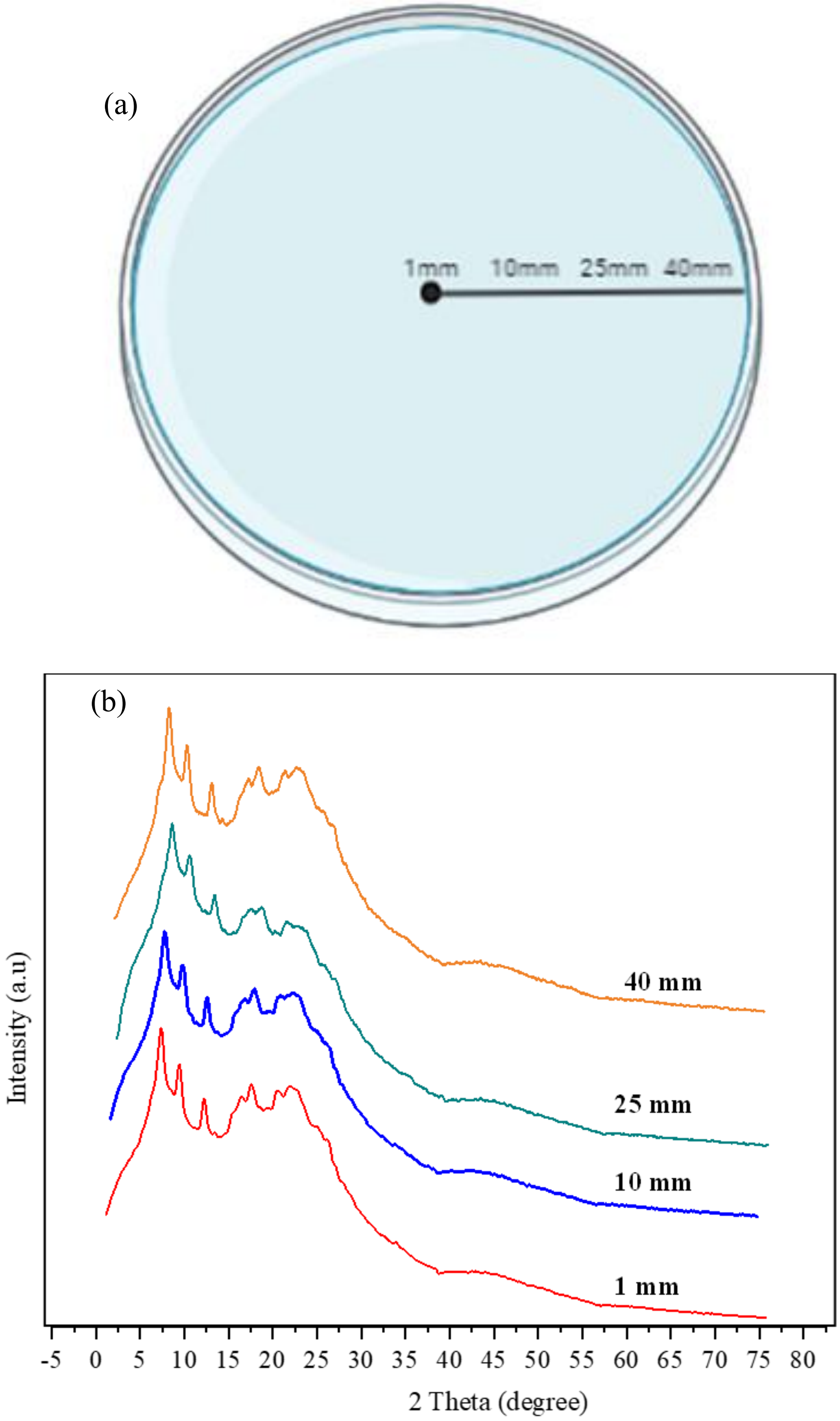

Fig. S3: (a) Pictorial view; (b) XRD spectrum of samples collected from different interval of the electro-casted membrane.

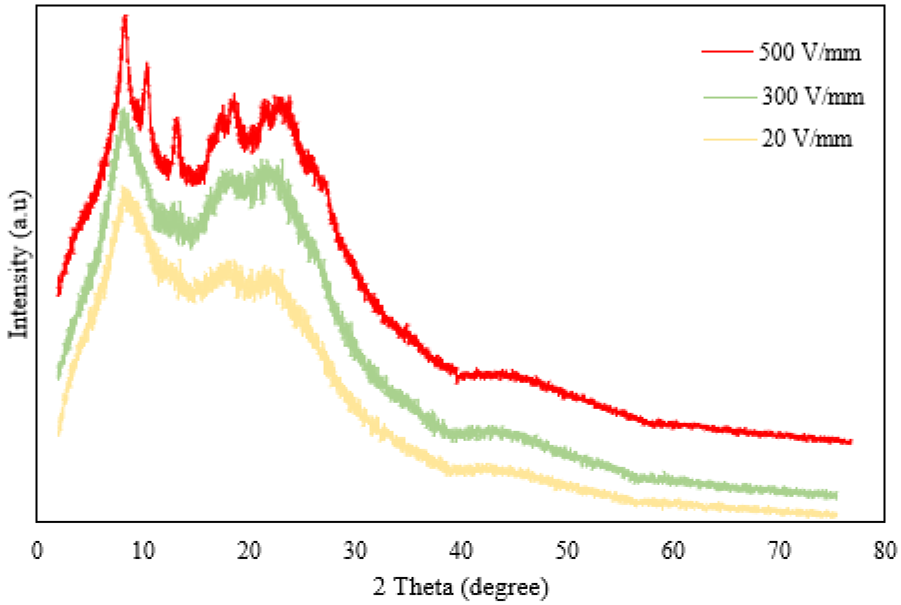

Fig. S4: XRD spectrum of the preliminary results for membranes obtained at different electric field strengths.

S1: Membrane Separation Performance

The separation performance of the membranes was evaluated by steadily increasing the upstream gas inlet pressure, while keeping the downstream at fixed volume. As a way of ensuring reproducibility of the results, the measurement was conducted three times, and the average value was reported. The permeability coefficient of the gas was computed using Equation 1, based on the values obtained from the slope along the steady-state region. The ideal selectivity for binary gas pairs of A and B was evaluated using Equation 2.

$$P = \frac{273.15 \times 10^{10}}{760} \frac{VL}{AT\Delta p}\left(\frac{dp}{dt}\right) \quad (1)$$

$$\alpha_{AB} = \frac{P_A}{P_B} \quad (2)$$

Where P is the permeability, in Barrer (1 Barrer =10^{-10} [cm^3(STP) cm] / (cm^2s cmHg)); V is the permeate volume (cm^3); L is the membrane thickness (cm); A is the effective membrane area (cm^2); T is the operating temperature (K); Δp *is* the pressure difference between the injection and

the permeate sides (cmHg); dp/dt is the steady state rate of pressure rise. P_A and P_B are the permeability coefficient of gases A and B, respectively. The more permeable gas is taken by default as the gas A, as such, $a_{AB} > 1$.

To determine the diffusion coefficient, D, of each gas through the membranes, the time lag method was utilized[1]. This involved using the equation which takes into account the membrane thickness (l) and the gas time lag (θ) according to Equation 3. By assuming the solution-diffusion transport mechanism was valid, the solubility coefficient, S, was indirectly obtained as a ratio of the permeability and the diffusion coefficient using Equation 4.

$$D = \frac{l^2}{6\theta} \tag{3}$$

$$S = \frac{P}{D} \tag{4}$$

Table S1: CA-IL-e membrane gas permeability and ideal gas selectivity at different ILs loading.

	Average permeability			Ideal gas selectivity	
**IL Loading (%)**	**N_2**	**CH_4**	**CO_2**	**CO_2/N_2**	**CO_2/CH_4**
**0.00**	0.5	0.7	8.5	17.96	12.36
**10.00**	0.7	0.9	8.9	13.62	10.17
**20.00**	0.9	1.2	12.6	14.76	10.24
**30.00**	1.2	1.8	19.0	15.51	10.47

S2: Membrane Thermal Analysis

The thermal stability analysis of the fabricated membranes was by thermogravimetric (TGA) and Differential Scanning Calorimetry (DSC). The thermogravimetric analysis was performed on both the pristine CA membrane and the composite CA-ILs membranes, which were fabricated using different concentrations of ILs, in an Argon atmosphere to assess their degradation behavior. The TGA curves presented in the analysis do not indicate any traces of moisture or residual solvent (Fig. 4). The TGA curve of the pristine CA membrane demonstrated stability without any

discernible weight loss up to 300 °C. However, the pure CA membrane's stability was considerably diminished beyond this temperature, as evidenced by weight loss from 100% to approximately 15% between 300 °C and 500 °C, as shown in Fig. 4a. Similarly, the thermogravimetric analysis of the pure [EMIM][DCA] IL indicated an initial weight loss at around 280 °C, with thermal degradation continuing from 100% to approximately 20% weight loss between 280°C and 800 °C.

In addition, Fig. 4b illustrates the thermal analysis of the composite CA-ILs membrane fabricated with and without applying an electric field at an IL loading of 40%. The composite membrane CA-IL40-e shows a modest enhancement in degradation temperature compared with CA-IL40 and this is due to the crystallinity reinforcement through applying the electric field to the membrane during the fabrication. This regular arrangement provides stability and strength to the membrane, making it less susceptible to thermal stress. Crystalline regions can act as physical barriers, hindering the diffusion of heat and preventing the propagation of thermal energy through the material [2]

The TGA for lower IL loading without applying an electric field is presented in Fig. 4a. The TGA curves of the composite CA-ILs exhibited similar characteristics, with initial degradation beginning at approximately 200 °C and continuous degradation from 100% to around 20% weight loss beyond this temperature. The figure shows that the thermal stability of CA-ILs was found to be lower than that of the pure CA membrane due to the presence of ILs in the CA polymer matrix, which is consistent with previous studies[3–6]. This observation may be attributed to the fact that the [EMIM][DCA] IL often starts to degrade before the polymer matrix, as shown in Fig. 4a, which correspondingly implies that the dissolved ILs affects the way the CA chains are arranged, making it easier to break the bonds between the polymer chains and making them more susceptible to degradation. However, it is important to note that the degradation pattern could vary depending on the rate at which temperature is increased[7].

The figure also reveals that the residual weight loss for both the pure IL and the corresponding CA-ILs is greater than that of the pristine CA membrane. This observation provides further evidence of the presence of IL in the polymer matrix and the residual weight loss is found to increase with increasing IL concentration. This finding aligns with previous research reported by [2].

The DSC analysis conducted to study the effect of blending IL in the polymer matrix on the flexibility of polymer chains and the miscibility of IL in the CA polymer matrix for both pure CA

and CA-ILs membranes are shown in Fig S5. The figure shows the glass transition (T_g), while Table S2 has the values of both the T_g and decomposition (T_d) of pristine CA and CA-ILs composite membranes. The T_g of the pristine CA membrane falls between 157 °C and 190 °C, while the T_d is between 200 °C and 250 °C, which is consistent with values reported by other researchers. The T_g of the composite CA-ILs membranes is lower than that of the pristine CA membrane, with a slight variation observed between the ones fabricated with and without an electric field at higher ILs loading. Due to the reduction of T_g of the membranes, they are expected to have higher permeability than pure CA membranes. The permeability of a polymer membrane is closely related to the mobility of its polymer chains. For instance, when a polymer is above its T_g, the increased mobility of the polymer chains allows for more accessible transport of molecules through the membrane. Therefore, a reduction in T_g generally results in increased permeability because the polymer chains are more mobile at a given temperature.

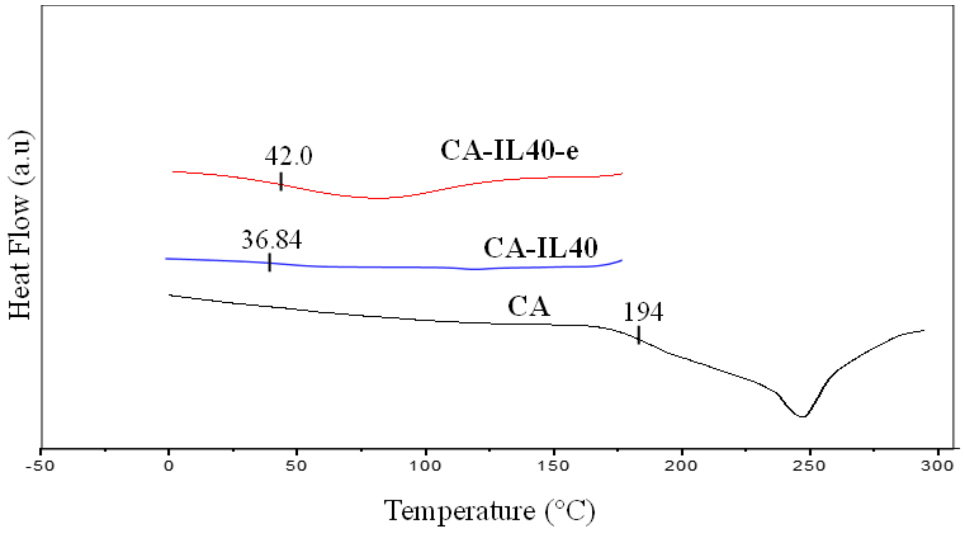

Fig. S5: DSC analysis for: Pure CA, CA-IL40 and CA-IL40-e.

Table S2: Glass temperature of CA-ILs membrane without an electric field

IL Loading (%)	T_g	T_d
**0.00**	194	303
**10.00**	140.3	212.42
**20.00**	102	210.51

**30.00**	61.96	208.8

S3: Effect of feed pressure on the CA-ILs membrane performance

The impact of feed pressure on the separation performance of the composite CA-ILs membrane fabricated using an electric field application, with higher IL loading of 40% is depicted in Fig. S6 and Fig. S7. The permeability of N_2, CH_4, and CO_2 gases were measured at various feed pressures, and it was observed that increasing feed pressure led to higher gas permeability for the membranes with the same IL loading (Fig S6). However, the permeability of CO_2 gas is notably higher than that of N_2 and CH_4 gas, primarily due to the smaller kinetic diameter of CO_2 and its strong affinity to the ILs. This finding is consistent with previous studies[4,8]. In addition, increasing feed pressure also resulted in higher pair gas (CO_2/N_2 and CO_2/CH_4) selectivity, as shown in Fig. S7. The maximum permeability and ideal selectivity were achieved at the highest feed pressure studied for all membranes. However, the increase both in permeability and ideal selectivity due to applied pressure is not significant. No differences in membrane thickness or weight were observed before and after the gas permeation experiments for all membranes tested, indicating the absence of any swelling or plasticization phenomena during the experiments. Plasticization is a phenomenon that depends on pressure and is characterized by a sudden increase in permeability and decrease in selectivity beyond a threshold pressure limit, referred to as plasticization pressure[9]. The absence of this behavior observed in the current study indicates that there was no occurrence of plasticization phenomena in the synthesized CA-ILs membranes.

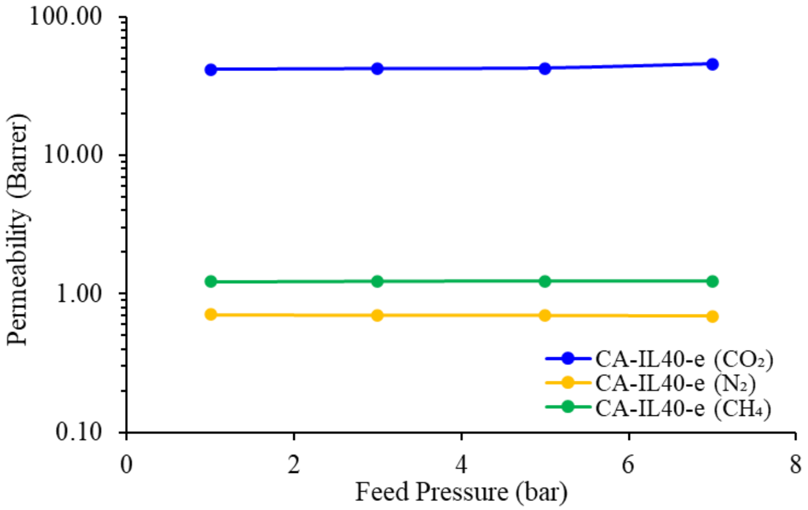

Fig. S6: Effect of feed pressure on single gas (CO_2, N_2 and CH_4)

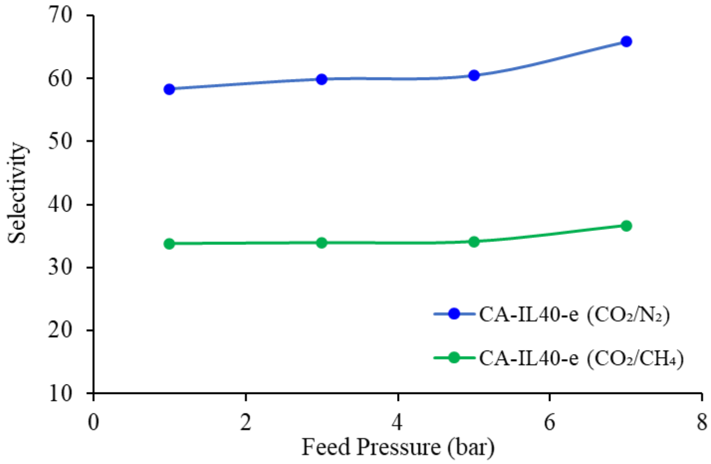

Fig. S7: Effect of feed pressure on gas selectivity (CO_2/N_2 and CO_2/CH_4).

S4: Membranes Stability

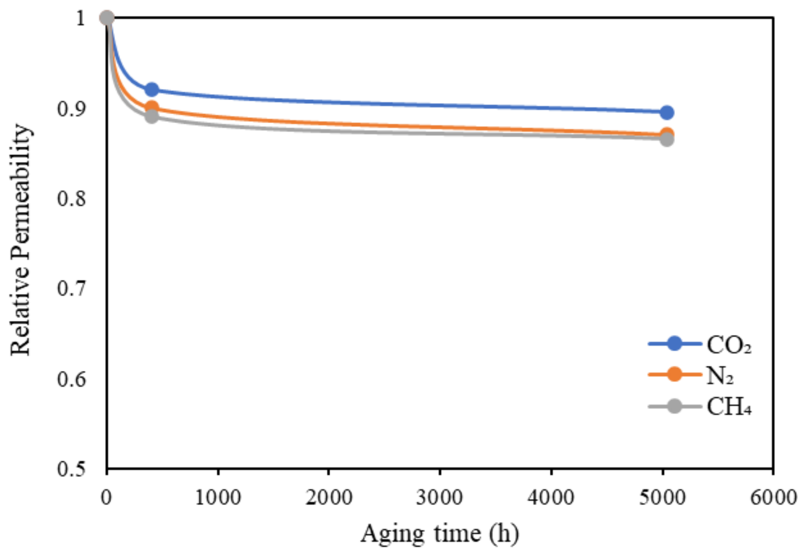

Fig. S8: Relative permeability variation profiles over time of CA-IL40-e.

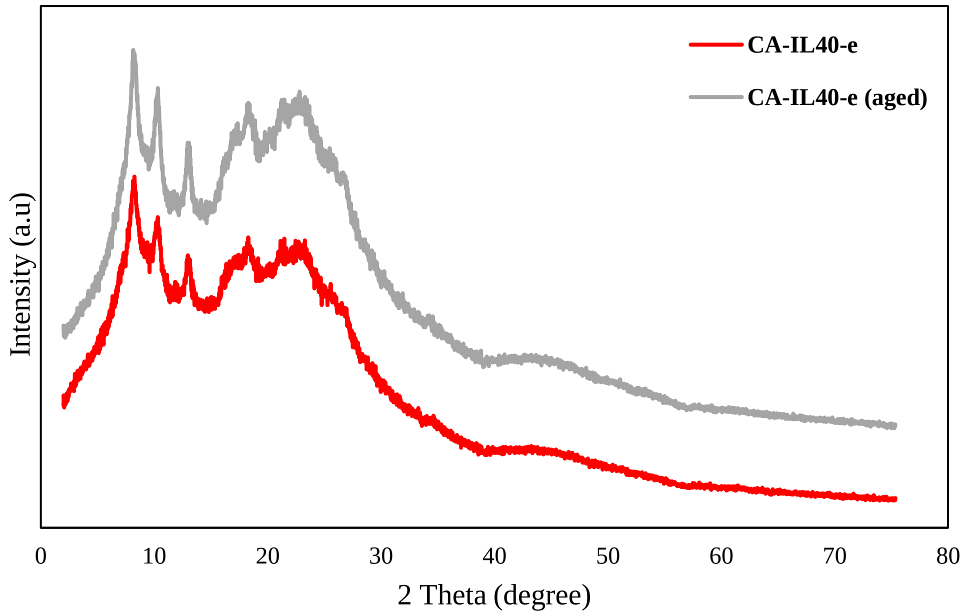

Fig. S9: XRD spectra of CA-IL40-e and aged CA-IL40-e (5040 h).

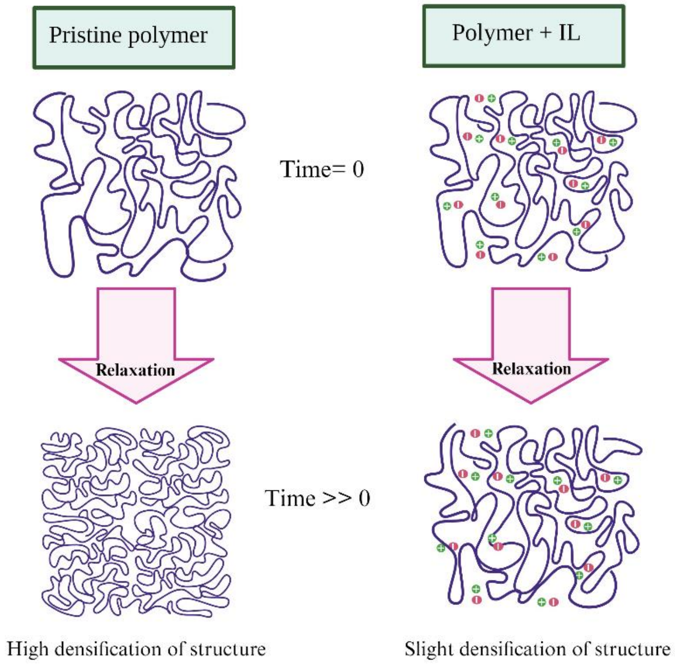

Fig. S10: Schematic illustrate pristine polymer and polymer+ IL structural changes over time.

S5: Electric Field Modelling

A Computational Fluid Dynamics (CFD) model was developed using COMSOL Multiphysics to visualize the electric field distribution imposed to the membrane during electro-casting. The model consisted of an axis symmetric model (half of the membrane), 2-dimensional model which employs the Finite Volume Method (FVM) to discretize the governing equations and solve for the electric field. Fig S11a depicts a schematic of the membrane/needle system while Fig. S11b shows the mesh generated.

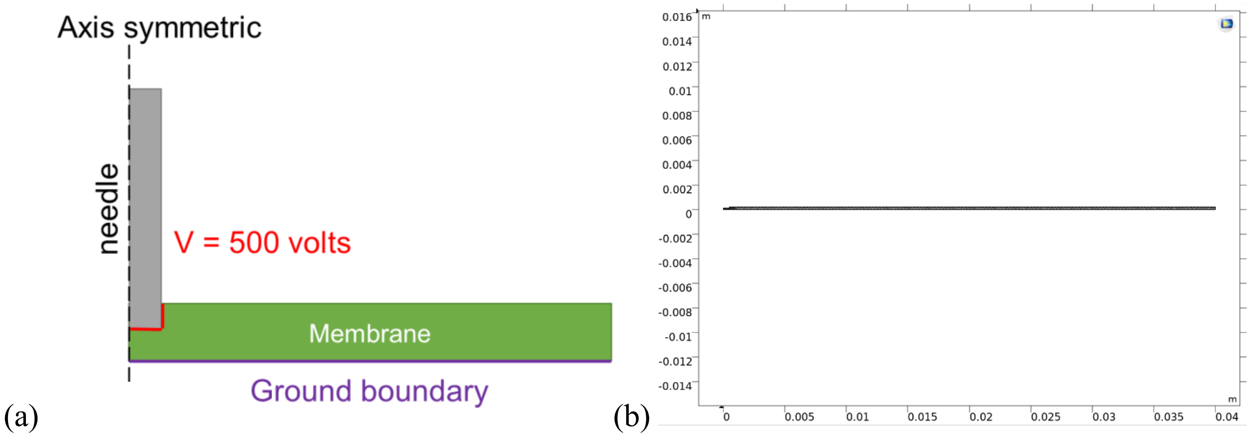

Fig. S11: (a) Schematic of the membrane/needle system (not at scale); and (b) Mesh of the axis symmetric CFD model (at scale), displaying only half of the membrane and the immersion of the needle (left)

The electrical conductivity of the polymer/IL system was selected as 0.708 – based on its composition and a relative permittivity of 18.The boundary conditions included electrical potential of 500 volts (Fig S11a: red line) and ground V=0 for the bottom plate (Fig S11a: purple line). Electrostatics was selected as the physics of the system and used to solve the following equations, where E represents the electric field, v the electric potential, ϵ_0 the permittivity of free space, ϵ_r the relative permittivity, and ρ_v the polarization vector.

$$E = -\nabla v$$

$$\nabla \cdot (\epsilon_0 \epsilon_r E) = \rho_v$$

Fig. S12 portrays that in the vicinity of the needle, the electric field exhibits high intensity and dissipates radially. By considering the end of the needle as the reference point, it can be observed that the radial electric field reaches zero after a distance of 0.0005 m.

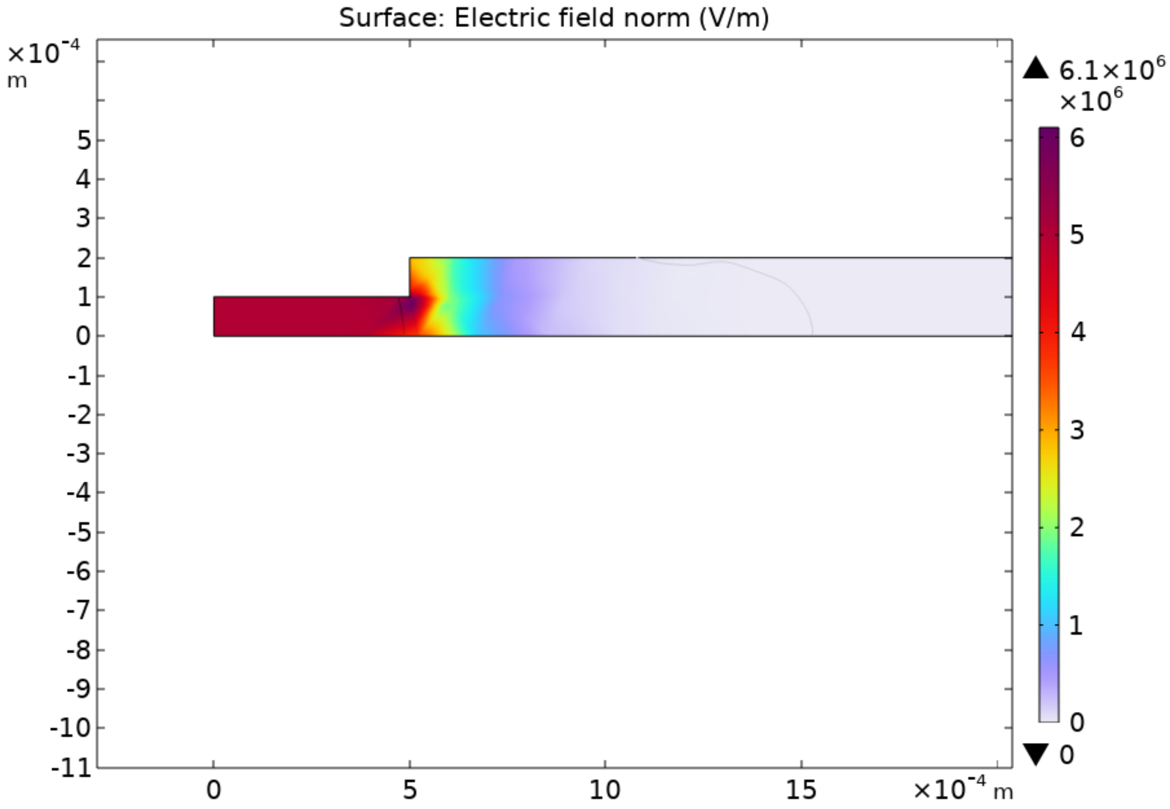

Fig. S12: Electric field distribution across the membrane

Reference

(1) Ye, C.; Wu, X.; Wu, H.; Yang, L.; Ren, Y.; Wu, Y.; Liu, Y.; Guo, Z.; Zhao, R.; Jiang, Z. Incorporating Nano-Sized ZIF-67 to Enhance Selectivity of Polymers of Intrinsic Microporosity Membranes for Biogas Upgrading. *Chem Eng Sci* **2020**, *216*.

(2) Chinyerenwa, A. C.; Wang, H.; Zhang, Q.; Zhuang, Y.; Munna, K. H.; Ying, C.; Yang, H.; Xu, W. Structure and Thermal Properties of Porous Polylactic Acid Membranes Prepared via Phase Inversion Induced by Hot Water Droplets. *Polymer (Guildf)* **2018**, *141*, 62–69.

(3) Kanehashi, S.; Kishida, M.; Kidesaki, T.; Shindo, R.; Sato, S.; Miyakoshi, T.; Nagai, K. CO2 Separation Properties of a Glassy Aromatic Polyimide Composite Membranes Containing High-Content 1-Butyl-3-Methylimidazolium Bis (Trifluoromethylsulfonyl)Imide Ionic Liquid. *J Memb Sci* **2013**, *430*, 211–222.

(4) Mannan, H. A.; Mohshim, D. F.; Mukhtar, H.; Murugesan, T.; Man, Z.; Bustam, M. A. Synthesis, Characterization, and CO2 Separation Performance of Polyether Sulfone/[EMIM][Tf2N] Ionic Liquid-Polymeric Membranes (ILPMs). *Journal of Industrial and Engineering Chemistry* **2017**, *54*, 98–106.

(5) Mohshim, D. F.; Mukhtar, H.; Man, Z. Ionic Liquid Polymeric Membrane: Synthesis, Characterization & Performance Evaluation. In *Key Engineering Materials*; Trans Tech Publications Ltd, 2014; Vol. 594–595, pp 18–23.

(6) Klepić, M.; Setničková, K.; Lanč, M.; Žák, M.; Izák, P.; Dendisová, M.; Fuoco, A.; Jansen, J. C.; Friess, K. Permeation and Sorption Properties of CO2-Selective Blend Membranes Based on Polyvinyl Alcohol (PVA) and 1-Ethyl-3-Methylimidazolium Dicyanamide ([EMIM][DCA]) Ionic Liquid for Effective CO2/H2 Separation. *J Memb Sci* **2020**, *597*.

(7) Lam, B.; Wei, M.; Zhu, L.; Luo, S.; Guo, R.; Morisato, A.; Alexandridis, P.; Lin, H. Cellulose Triacetate Doped with Ionic Liquids for Membrane Gas Separation. *Polymer (Guildf)* **2016**, *89*, 1–11.

(8) Seng, L. K.; Masdar, M. S.; Shyuan, L. K. Ionic Liquid in Phosphoric Acid-Doped Polybenzimidazole (Pa-Pbi) as Electrolyte Membranes for Pem Fuel Cells: A Review. *Membranes*. MDPI October 1, 2021.

(9) Nasir, R.; Mukhtar, H.; Man, Z.; Dutta, B. K.; Shaharun, M. S.; Abu Bakar, M. Z. Mixed Matrix Membrane Performance Enhancement Using Alkanolamine Solution. *J Memb Sci* **2015**, *483*, 84–93.
